# Bibliographical

**Published:** 1873-08

**Authors:** 


### BIBLIOGRAPHICAL.

Clinical Reports from Private Practice. By John Herbert Claiborne, A.M., M.D., one of the Vice-Presidents of the Medical Society of Virginia; lately Surgeon in the Provisional Army of the Confederate States, etc., etc. Petersburg, Virginia: Jos. Van Holt, Publisher. 1873.

Some months ago, our readers were notified of the preparation of the Clinical Reports of Dr. Claiborne, and we have now the pleasure of a copy on our table. Clinical reports of cases in actual practice, when well and succinctly written, photographs upon the mind the symptoms and treatment of human maladies most vividly and indelibly. With a few strokes of the pencil of the true artist, the landscape grows into almost living reality before the eye, and so, too, when an author of the skill and literary ability of Dr. Claiborne sketches before the mental eye his vivid bedside pictures of diseases, and the treatment instituted for the relief of his patients, we feel something of the inspiration moving the heart and soul of the great physician, and enter into the fears and hopes of that treatment upon which the life of a fellow-mortal seems suspended. In no other manner can a great teacher so graphically paint the maladies preying upon our mortal frames than by well-prepared clinical reports, and our author has succeeded in presenting his brethren a volume of very great interest and practical value. To the Southern practitioner, especially, it is of the highest value, giving reports of cases occurring in our climate, and among patients such as they are daily called to visit and prescribe for.

The volume abounds with interesting cases, among the number of which we find: Nephritic colic; irritable bladder; dysmenorrhœa; asoturia; hemorrhoids; hematuria; hemorrhage; scarlet fever; albuminuria; rheumatism; neuralgia; acute ovaritis; pneumonia; poisoning by strychnia; epidemic catarrh; hepatitis; weed; acute eczema; chronic eczema; cholera infantum; cancer of the womb; induration cervix uteri; paralysis and epilepsy; chorea; infantile convulsions; sciatica; sore throat; measles; jaundice; ulceration cervix; uterine hemorrhage; follicular diphtheritis; chronic intermittent fever; bronchitis; camp itch; vomiting; debility; orchitis; gout; urticaria; periodic *vs.* typhoid fever; remittent fever; nervous prostration; menorrhagia, etc., etc.—forming a most interesting and instructive volume. The treatment, forming the ground mainly of these reports, illustrates the large experience, and attests the scientific skill of a thoroughly-read and close-observing physician. It is a volume at once creditable to our section and to the profession at large. We trust it will meet with the appreciative favor of the profession every where, and of Southern practitioners especially. We most cordially commend it to our readers, and express the hope that each may secure a copy for study and reference; and also that our author may receive that encouragement which shall induce him very shortly to issue the second volume promised.

**Report of Columbia Hospital for Women and Lying-in Asylum, Washington, D. C. By J. Harry Thompson, A.M., M.D., Surgeon-in-Chief. With an Appendix.**

This is a large and handsome volume. The Report is highly creditable to the government and the distinguished surgeons and physicians connected with Columbia Hospital. A clinical record of some seven hundred cases is given, with illustrations of interesting and important cases. Thirty-four successive operations for the radical cure of lacerated or ruptured perineum, resulting favorably, at the very threshold of the Report arrest the attention; and further on, "vesico-vaginal and recto-vaginal fistula," "vaginal rectocele," "diseases and displacements of the uterus," "chronic cervical metritis and endometritis," "pelvic cellulitis," etc., give the reader ample scope for study and reflection.

The appendix, containing reports from the dispensary connected with the hospital, is not a whit behind the former in interest. F. A. Ashford, M.D , Department of the Diseases of Women; Samuel C. Busey, M.D., Department of the Diseases of Children; and D. Webster Prentiss, M.D., Department of the Diseases of the Eye and Ear, each give reports highly flattering to the hospital and to the respective authors.

We return thanks for the volume, and hope many of our readers may secure a volume—one of the largest and most scientific ever issued from any American hospital.

